# Supplementary material for: Sequencing and Structural Analysis of the Complete Chloroplast Genome of the Medicinal Plant Lycium chinense Mill
Source: Plants (Basel). 2019 Apr 3;8(4):87. doi: 10.3390/plants8040087 (PMC6524360; doi:10.3390/plants8040087)
Supplement: Supplementary file 1 [file plants-08-00087-s001.pdf]

Table S1. Codon usage in the *L. chinense* chloroplast genome.

Amino Acid	Codon	Num.	Total	Frequency	Amino Acid	Codon	No.	Total	Frequency
Asp	GAT	400	613	2.31%	Lys	AAA	993	1,666	6.27%
	GAC	213				AAG	673		
Leu	TTA	637	3,496	13.16%	Glu	GAA	645	1,029	3.87%
	TTG	796				GAG	384		
	CTT	615			Thr	ACT	200	951	3.58%
	CTC	433				ACC	207		
	CTA	563				ACA	340		
	CTG	452				ACG	204		
Ile	ATT	839	2,341	8.81%	Ala	GCT	138	606	2.28%
	ATC	690				GCC	149		
	ATA	812				GCA	194		
Met	ATG	731	731	2.75%		GCG	125		
Asn	AAT	670	1,093	4.11%	Tyr	TAT	676	1,061	3.99%
	AAC	423				TAC	385		
Val	GTT	455	1,525	5.74%	His	CAT	310	556	2.09%
	GTC	281				CAC	246		
	GTA	446			Cys	TGT	266	484	1.82%
	GTG	343				TGC	218		
Phe	TTT	904	1,704	6.41%	Trp	TGG	496	496	1.87%
	TTC	800			Arg	CGT	101	1,423	5.36%
Ser	TCT	428	1,758	6.62%		CGC	94		
	TCC	284				CGA	231		
	TCA	428				CGG	238		
	TCG	310				AGA	461		
	AGT	189				AGG	298		
	AGC	119			Gly	GGT	253	1,169	4.40%
Pro	CCT	231	1,030	3.88%		GGC	184		
	CCC	208				GGA	365		
	CCA	311				GGG	367		
	CCG	280			Stop	TAA	604	1,862	7.01%

Gln	CAA	567	975	3.67%	TAG	676
	CAG	408			TGA	582

Table S2. RNA editing predicted in the chloroplast genomes of *L. chinense* by the PREP program.

Gene	Nucleotide Position	Codon Change	Amino Acid Change	Score
accD	/	/	/	/
atpA	791	CCC-CTC	P-L	1
atpB	/	/	/	/
atpF	157	CCG-TCG	P-S	1
atpI	/	/	/	/
ccsA	/	/	/	/
clpP	296	ACT-ATT	T-I	1
	524	GCC-GTC	A-V	1
matK	/	/	/	/
ndhA	8	ACA-ATA	T-I	1
	302	ACA-ATA	T-I	1
	326	GCA-GTA	A-V	1
	596	CCT-CTT	P-L	1
	1,037	CCC-CTC	P-L	1
ndhB	149	TCA-TTA	S-L	1
	467	CCA-CTA	P-L	1
	586	CAT-TAT	H-Y	1
	611	TCA-TTA	S-L	0.8
	737	CCA-CTA	P-L	1
	746	TCT-TTT	S-F	1
	830	TCA-TTA	S-L	1
	836	TCA-TTA	S-L	1
	1,481	CCA-CTA	P-L	1
ndhD	2	ACG-ATG	T-M	1
	383	TCA-TTA	S-L	1
	674	TCG-TTG	S-L	1
	878	TCA-TTA	S-L	1
	1,298	TCA-TTA	S-L	0.8

	1,310	TCA-TTA	S-L	0.8
ndhF	290	TCA-TTA	S-L	1
ndhG	/	/	/	/
petB	418	CGG-TGG	R-W	1
	611	CCA-CTA	P-L	1
petD	368	TCC-TTC	S-F	1
petG	/	/	/	/
petL	/	/	/	/
psaB	/	/	/	/
psaI	/	/	/	/
psbB	/	/	/	/
psbE	214	CCT-TCT	P-S	1
psbF	/	/	/	/
psbL	2	ACG-ATG	T-M	1
rpl2	164	ACT-ATT	T-I	1
	206	ACC-ATC	T-I	1
rpl20	308	TCA-TTA	S-L	0.86
rpl23	/	/	/	/
rpoA	830	TCA-TTA	S-L	1
rpoB	338	TCT-TTT	S-F	1
	473	TCA-TTA	S-L	0.86
	551	TCA-TTA	S-L	1
	2,000	TCT-TTT	S-F	1
	2,426	TCA-TTA	S-L	0.86
rpoC1	407	TCA-TTA	S-L	0.86
	485	CCC-CTC	P-L	0.86
	973	CCG-TCG	P-S	1
	1,019	GCA-GTA	A-V	0.86
	1,250	GCA-GTA	A-V	0.86
	1,414	CTT-TTT	L-F	1
	1,847	TCG-TTG	S-L	1
rpoC2	2,287	CGG-TGG	R-W	1
	3,731	TCA-TTA	S-L	0.86
rps12	248	TCA-TTA	S-L	1

rps8	/	/	/	/
rps14	80	TCA-TTA	S-L	1
rps16	/	/	/	/
ycf3	/	/	/	/

Table S3. Chloroplast genomes used to study their phylogeny relationship in this study.

**Accession Number**	**Source Species**	**Family**
	*Lycium chinense.*Mill	Solanaceae
MH032560	*Lycium barbarum*	
NC_039651	*Lycium ruthenicum*	
NC_004561.1	*Atropa belladonna*	
NC_018552.1	*Capsicum annuum*	
NC_028007.1	*Capsicum frutescens*	
NC_018117.1	*Datura stramonium*	
NC_024261.1	*Hyoscyamus niger* L.	
MH019241.1	*Physalis angulata* L.	
NC_030282.1	*Scopolia parviflora*	
NC_008096.2	*Solanum tuberosum* L.	
NC_028069.2	Solanum commersonii	
NC_007898.3	*Solanum lycopersicum*	
NC_036733.1	*Przewalskia tangutica*	
NC_035233.1	*Salvia japonica* Thunb	Labiatae
KX230834.1	*Pogostemon cablin* (Blanco) Bent.	
